# To Weed or Not to Weed: A Systematic Review Exploring the Potential for Cannabis Use in Cardiovascular Disease, Mental Health and Pain Management

**DOI:** 10.7759/cureus.40606

**Published:** 2023-06-18

**Authors:** Kofi D Seffah, Mahendar Kumar, Namballa Naveen, Vamsi Krishna Pachchipulusu, Yubraj Paudel, Anandkumar Patel, Beenish Najam, Heet N Desai, Janan Illango, Pousette Hamid

**Affiliations:** 1 Internal Medicine, California Institute of Behavioral Neurosciences and Psychology, Fairfield, USA; 2 Internal Medicine, Piedmont Athens Regional Medical, Athens, USA; 3 Anaesthesia, Royal College of Surgeons in Ireland, Drogheda, IRL; 4 Internal Medicine, Steel Authority of India (SAIL) Hospital, Dhanbad, IND; 5 Research, California Institute of Behavioral Neurosciences and Psychology, Fairfield, USA; 6 Neurology, Shalby Hospitals Naroda, Ahmedabad, IND; 7 Medicine, Maharshi Hospital Private Limited, Surendranagar, IND; 8 Neurology, California Institute of Behavioral Neurosciences and Psychology, Fairfield, USA

**Keywords:** pain management, analgesic ladder, flavonoids, cardiopulmonary function, cardiovascular disease, weed, cannabis, medical cannabis, thc, cbd

## Abstract

Despite its historical reputation as a substance of abuse, cannabis use has increased following decriminalization efforts in the United States. It has historically garnered a bad reputation as a substance of abuse, but paradoxically is associated with an improved perception of well-being. We were interested in positive cardiovascular outcomes, both positive and negative mental health outcomes and impact on physical activity of cannabis, both recreational and medical. Databases included PubMed, ResearchGate, Cochrane, Science.gov and ScienceDirect. We were interested in cardiovascular, mental health and physical health in our search. Data included articles published during or after 2017. Our studies showed no cardiovascular benefits, increased risk of documented cardiovascular events and increased mortality associated with cannabis use. Physical benefits derived were largely in patients with chronic pain. With regards to mental health, the impact of the drug appears to be both positive and negative, with no clear benefits as a first-line agent. Route of administration appears to have an impact on the overall extent of side effects. Overall, medical cannabis appears to pose an almost negligible side effect profile compared to recreational. Our findings suggest that while cannabis use may offer benefits for chronic pain management, it is associated with increased cardiovascular risks. Further, medical cannabis appears to have a more favorable side effect profile compared to recreational use.

## Introduction and background

Historically, cannabis was first identified in Asia around 500 BC and was initially used medicinally. Uses have anecdotally ranged from the management of diarrhea and dyspepsia to anorexia. The plant has non-medical uses, including recreational, textile and in manufacturing [1,2]. In the United States, the plant has gone through several cycles of sociopolitical and legal repositioning, making it an illegal substance for much of the 20th century, but recently seeing progressive decriminalization in a majority of states [3]. Globally, an estimated 3.8% of the world population used cannabis around 2017 [1]. Twelve percent of people aged 12 and over have been exposed to cannabis in one form or another in the United States [2]. It however continues to be classified as a Schedule 1 drug by the Drug Enforcement Administration (DEA), meaning it has no health benefits and possesses a high risk of harm [3-5].

Medical marijuana has however seen a resurgence in the past two decades [1]. Tetrahydrocannabinol (THC) is known for psychoactivity whilst cannabidiol (CBD) is marketed for its anti-inflammatory properties, with current medicinal recommendations for the management of cancer-related anorexia, nausea and vomiting, chronic pain, and as an adjunct in modulating neurodivergent behavior [1]. No deaths from recreational or medicinal use of the plant or drug have been reported [3,4].

Currently, it is known that the plant is of healthcare and economic importance, both positive and negative. Anecdotally, both medicinal and recreational use have been associated with increased physical output, pain relief, improvement in anxiety and other mood disorders. It possesses the potential for abuse, however [6]. It is still considered by some as a gateway drug [3].

Whilst we take cognizance of these factors, we seek to find out if there is any evidence of cardiovascular benefit, either for the prevention of heart disease or the management of pre-existing ones. Secondly, we seek to find out if there is evidence for improved physical activity with the use of cannabis. Finally, our research seeks to explore if there are any therapeutic mental health benefits to the use of the drug, that would foster more research into its use as a first-line agent for the management of any condition. 

## Review

Methods

We conducted our review in accordance with the directives enshrined in the Preferred Reporting Items for Systematic Reviews and Meta-Analyses (PRISMA) guidelines [7].

Inclusion and Exclusion Criteria

All individuals, regardless of age, sex, ethnicity or background who had used any form of cannabis, including medical and recreational cannabis, were included in the study. Meta-analyses, systematic reviews, case reports and observational studies were accepted as part of our criteria. All articles had to be published in English or had to have been translated from another language into English. Cardiovascular, physical and mental health outcomes were the focus of our search. Articles addressing these items were selected for quality review. With previously established proclivity of cannabis and mental health research, we chose to highlight our search criteria on the other areas of interest - cardiopulmonary and physical health, whilst acknowledging mental health effects and conclusions drawn from our chosen studies. Research had to have been conducted on or after 2017. We did not accept abstracts, textbook chapters, opinion pieces, articles in other languages apart from English, and articles published before 2017. Articles that addressed other areas of health, but excluding mental or cardiovascular health were excluded. As much as possible, we excluded studies that made cannabis only one of several drugs of addiction or one of several drugs under study, as we wanted to eliminate confounders and maintain our focus. Table 1 below highlights and summarizes the inclusion and exclusion.

**Table 1:** Inclusion and Exclusion Criteria for Publication Selection The terms cannabis and marijuana were used interchangeably

Inclusion criteria	Exclusion criteria
All individuals who have used a form of cannabis	Articles comparing cannabis and other substance of abuse
Full text articles only	Abstracts, opinion texts
Written in English	Non-English text/article
Written on or after 2017	Written before 2017
Must address cardiovascular and mental health	Excludes cardiovascular or mental health in its assessment

Search Strategy

From April 4th to April 11th 2023, we searched PUBMED, including the Medical Subject Heading, ResearchGate, Cochrane Reviews, Science.gov and Science.com for articles using keywords such as CBD, THC, Medical cannabis, cannabis, weed, cardiovascular disease, pulmonary disease, cardiopulmonary function, flavonoids, analgesic ladder, benefits, harms, health, first line, long-term use, WHO (World Health Organization) analgesic ladder, guideline, improvement, physical functioning and well-being. Our keywords were combined with the Booleans ‘AND’ and ‘OR’. The conclusive list of databases with search strategies is noted in Table 2 below. Manual searches were conducted to supplement our efforts by searching other databases like the DEA website.

**Table 2:** Databases, Search Strategy and Tally of Publications Selected MeSH Medical Subject Heading

Database	Search Strategy	Number of publications reviewed
PUBMED	Medical cannabis AND cardiovascular disease AND pulmonary disease AND pain	23
PUMBED Mesh	((("Cannabis"[Mesh]) AND "Pain"[Mesh]) AND "Cardiovascular Diseases"[Mesh]) AND "Lung Diseases"[Mesh] OR ((( "Medical Marijuana/adverse effects"[Mesh] OR "Medical Marijuana/history"[Mesh] OR "Medical Marijuana/poisoning"[Mesh] OR "Medical Marijuana/therapeutic use"[Mesh] )) AND "Heart Diseases"[Mesh]) AND "Physical Conditioning, Human"[Mesh]	1
Cochrane	cannabis AND heart AND lungs AND pain AND therapy	3
ScienceDirect	therapeutic cannabis AND marijuana use AND cardiovascular disease treatment AND pulmonary disease treatment AND pain AND benefits AND harms AND mortality	50
Science.gov	therapeutic cannabis AND marijuana use AND cardiovascular disease treatment AND pulmonary disease treatment AND pain AND benefits AND harms AND mortality	6
ResearchGate	therapeutic cannabis AND marijuana use AND cardiovascular disease treatment AND pulmonary disease treatment AND pain AND benefits AND harms AND mortality AND physical functioning	100

Selection Process

We manually evaluated each study, initially paying attention to data that met our inclusion criteria. Duplicates were promptly excluded at this phase. We screened 188 articles and took out 35 duplicates. We excluded a further 153 for not meeting our initial search criteria. We sought to individually review the remaining 34, but for four of them, full-text articles could not be obtained. The remaining articles were reviewed using quality assessment tools. Of these, five met our pre-determined cut-off of 60% or more, met our inclusion criteria and satisfied the demands of this study.

Results

**Figure 1:**
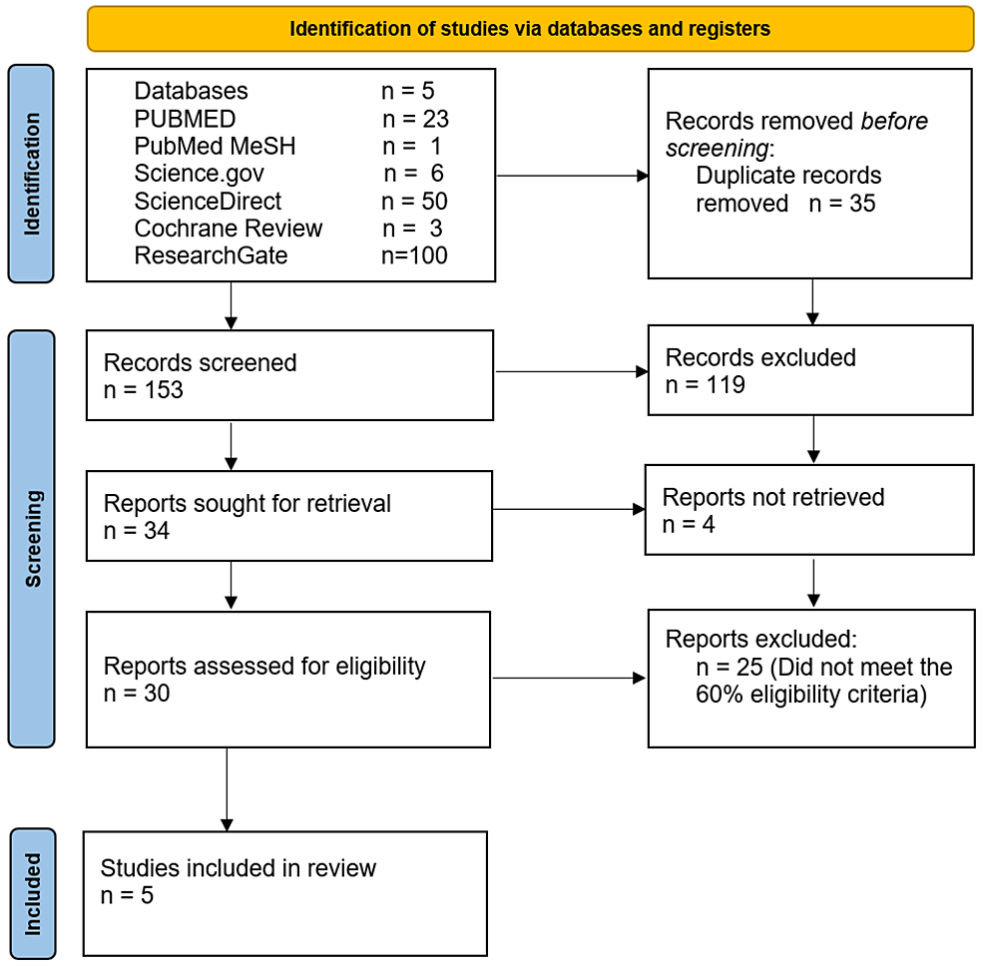
PRISMA Identification of Studies PRISMA - Preferred Reporting Items for Systematic Reviews and Meta-Analyses; MeSH - Medical Subject Heading

Of the five databases screened, five articles out of 188 remained for our evaluation and review. These include one meta-analysis, two systematic reviews, a cohort study and one cross-sectional study. A headcount of more than 450,000 patients were involved in the study, with the greatest contribution coming from our chosen cross-sectional survey. Two of our studies were interested in both cardiovascular and mental health outcomes, two in cardiovascular outcomes alone and one focused on mental health matters alone. The full search strategy is outlined in Figure 1 below.

We identified three studies, consisting of one meta-analysis and two systematic reviews. A Measurement Tool to Assess systematic Reviews (AMSTAR) was employed in evaluating the pre-determined quality cut-off of 60%. Table 3 below highlights the components of this assessment.

**Table 3:** AMSTAR Criteria Used in Quality Assessment of Selected Meta-Analysis and Systematic Reviews AMSTAR - A Measurement Tool to Assess systematic Reviews; PRISMA - Preferred Reporting Items for Systematic Reviews and Meta-Analyses; MeSH - Medical Subject Heading; PICO - Patient/Problem, Intervention, Comparison and Outcome

AMSTAR criteria	Latif [1]	Kennedy [2]	Wang [8]
Did the research questions and inclusion criteria for the review include the components of PICO?	Yes	Yes	Yes
Was a “priori” design implemented?	Yes	Yes	Yes
Did the review authors explain their selection of the study designs for inclusion in the review?	Yes	Yes	Yes
Did the review authors use a comprehensive literature search strategy?	Yes	Yes	Yes
Did the review authors perform study selection in duplicate?	No	No	Yes
Did the review authors perform data extraction in duplicate?	Uncertain	No	Yes
Did the review authors provide a list of excluded studies and justify the exclusions?	No	No	No
Did the review authors describe the studies included in adequate detail?	Yes	Yes	Yes
Did the review authors use a satisfactory technique for assessing the risk of bias in individual studies that were included in the review?	Yes	No	Yes
Did the review authors report on the sources of funding for the studies included in the review?	Yes	Yes	Yes
If a meta-analysis was performed, did the authors use appropriate methods to statistically combine results?	No meta-analysis	No meta-analysis	Yes
If a meta-analysis was performed, did the review authors assess the potential impact of risk of bias in individual studies on the results of the meta-analysis or other evidence synthesis?	No meta-analysis	No meta-analysis	Yes
Did the review authors account for risk of bias in individual studies when interpreting/discussing the results of the review?	Yes	Yes	Yes
Did the review authors provide a satisfactory explanation for and discussion of any heterogeneity observed in the results of the review?	Yes	Yes	Yes
If they performed quantitative synthesis, did the review authors carry out an adequate investigation of publication bias (small study bias) and discuss its impact on the results of the review?	No quantitative analysis performed	No quantitative analysis performed	Yes
Did the review authors report any potential sources of conflict of interest, including any funding they received for conducting the review?	Yes	Yes	Yes
Total score (out of 16)	10/13	9/13	15/16
Overall methodological quality	Accepted 76.9%	Accepted 69.2%	Accepted 93.75%

One cohort study was identified using our search criteria and the Joanna Briggs Institute critical appraisal checklist was used in determining suitability for further review. Table 4 below summarizes these findings.

**Table 4:** JBI Critical Appraisal Checklist for Cohort Studies JBI - Joanna Briggs Institute

Author: Zongo [9] Year: 2022	
Quality appraisal question	Answer
1. Were the two groups similar and recruited from the same population?	Yes
2. Were the exposures measured similarly to assign people to both exposed and unexposed groups?	Yes
3. Was the exposure measured in a valid and reliable way?	Yes
4. Were confounding factors identified?	Yes
5. Were strategies to deal with confounding factors stated?	No
6. Were the groups/participants free of the outcome at the start of the study (or at the moment of exposure)?	No
7. Were the outcomes measured in a valid and reliable way?	Yes
8. Was the follow up time reported and sufficient to be long enough for outcomes to occur?	Yes
9. Was follow up complete, and if not, were the reasons to loss to follow up described and explored?	Yes
10. Were strategies to address incomplete follow up utilized?	No
11. Was appropriate statistical analysis used? Overall appraisal: Accept	Yes

 We identified a single cross-sectional study, employing the NewCastle Ottawa Classification Tool for cross-sectional studies in quality appraisal. This study subsequently satisfied our search criteria, as highlighted in Table 5 below.

**Table 5:** NewCastle Ottawa Classification Tool for Quality Assessment of Cross-Sectional Studies * Indicates extent of quality per tool requirements

Article	Representativeness *	Sample size *	Non-respondents *	Ascertainment of exposure **	The subjects in different outcome groups are comparable, based on the study design or analysis. Confounding factors are controlled. **	Assessment of outcome *****	Statistical test *	Accept/Reject
Desai [10]	*	*	*	**	**	****	*	Accept

Study Characteristics

Five studies were selected that studied cannabis as a primary focus. Of these, two were systematic reviews, both of which addressed mainly physical conditioning and cardiovascular outcomes [1,2]. The meta-analysis involved in the study was interested in chronic pain patients using medical cannabis, and in finding out, broadly, and with evidence, the myriad of outcomes, both positive and negative, associated with the prescribed form [8]. The emphasis of the cohort study included was on medical cannabis as well, highlighting its role in emergency admissions for cardiovascular and mental health purposes [9]. Conversely, the cross-sectional study involved highlighted recreational use effects, noting increased cardiac and mental health incidents amongst its users. Table 6 below summarizes our findings for each of the articles chosen for this review.

**Table 6:** Summary of Study Characteristics Three systematic reviews, one cohort study and one cross-sectional study

Type of paper	Title	Authors	Year of Publication	Number of patients/articles reviewed	Summary of cardiovascular findings	Summary of mental health findings
Systematic review	The Impact of Marijuana on the Cardivascular System: A Review of the Most Common Cardiovascular Events Associated with Marijuana Use - PubMed (nih.gov)	Zara Latif [1]	2020	92 articles	Adverse outcomes of cannabis use with cardiovascular health	
Systematic review	Cannabis: Exercise performance and sport. A systematic review	Michael C Kennedy [2]	2017	15 articles	No improvement with aerobic performance and greater incidence of angina at a lower work load	
Systematic review	Medical cannabis or cannabinoids for chronic non-cancer and cancer related pain: a systematic review and meta-analysis of randomised clinical trials	Li Wang [8]	2021	32 trials with 5174 adult patients	Medical cannabis improves physical functioning	Medical cannabis improves chronic pain
Cohort study	Incidence and Predictors of Cannabis-Related Poisoning and Mental and Behavioral Disorders among Patients with Medical Cannabis Authorization: A Cohort Study	Arsene Zongo [9]	2022	29,153 individuals		Medical cannabis poses low poison and mental health risk in those authorized for medicinal purposes
Cross-sectional study	Primary Causes of Hospitalizations and Procedures, Predictors of In-hospital Mortality, and Trends in Cardiovascular and Cerebrovascular Events Among Recreational Marijuana Users: A Five-year Nationwide Inpatient Assessment in the United States	Rupak Desai [10]	2018	465,959 hospitalizations	Increased cardiovascular and cerebrovascular events amongst recreational users	Increased psychiatric admissions and procedures amongst recreational users

Discussion

Cannabis and Cardiovascular Health

The plant has had a role in performance enhancement for centuries, with undefined roles in stamina, relaxation and appetite stimulation [2]. This appears to be anecdotal, or, perhaps, an extrapolation of the mood benefits of the drug. One study [1,2] found that there were more cardiovascular events associated with cannabis use and exercise than otherwise. The drug was noted to cause tachycardia and sustained blood pressure lasting as long as three hours after recreational use [1,5]. Myocardial infarction has been reported in otherwise healthy individuals using the drug recreationally [1]. Indeed, a significant 1.2% of non-psychiatric diagnoses have been reported at discharge [10]. Other events including cardiomyopathies, cerebrovascular accidents, arteritis and sudden death have also been reported [1,2]. One explanation for the effect of marijuana on the heart could possibly be increased cardiovascular oxygen demand whilst using the drug [1,2]. Marijuana has been associated with increased atherosclerotic cardiovascular risk and thrombosis [1]. And whilst the drug may trigger a myriad of heart diseases, co-existence of these very conditions in recreational marijuana users is associated with a higher mortality [9].

Cannabis and Mental Health

Adverse events from cannabis, in the domain of mental health, are largely neurotic in nature [2]. The most common psychiatric diagnoses at discharge amongst recreational users are mood disorders, constituting 20.6% [10]. These adverse effects seem largely a product of recreational use [8]. Medical cannabis appears to enjoy a robust safety profile overall [9,10]. There also appears to be a relationship between the adverse events and the route of administration [1]. The issue of the drug causing addiction remains a matter up for debate, with the preferred terminology being ‘dependence’ and emphasis on frequency of use versus interference with activities of daily living [11]. There also appears to be a trend of said dependence amongst recreational users, as opposed to therapeutic use [11]. Psychoses are also impacted by cannabis use [12]. Age of onset of use, acute versus chronic use, pre-existing psychotic illness, and family history, amongst other factors, appear to determine the degree of psychosis or psychotic illness [12,13]. Conversely, people living with mental health are also more likely to use the drug recreationally [13,14]. There are arguments that cannabis may help with sleep, reduce anxiety and promote relaxation [15]. Findings however appear to be mixed and inconsistent in studies evaluated under this banner [6,9].

Cannabis and Pain Management

Non-inhaled medical cannabis has been shown to benefit chronic pain patients and offers some benefits with regard to their sleep quality. Medical cannabis has also been shown to improve physical functioning in this subset of people [8]. These benefits have however not been studied beyond a period of more than 5.5 months [8]. And compared with pre-existing modalities for the management of chronic pain including opioids, there are mixed reports, with one study showing no difference (mean deviation −0.13cm on 10cm visual analog scale for pain, −1.04 to 0.77cm) [8]. Even in the safest medical settings, side effects were noted. Cognitive impairment was noted amongst the subset of patients with chronic pain, and there was no improvement in social functioning, role functioning or emotional functioning [8]. Risk factors for the development of cannabis adverse effects include pre-existing mental health, pulmonary disease and diabetes [9]. Despite this seeming abundance of evidence leaning in favor of medical cannabis, and the emphasis of less utility for pain with recreational use, there continue to be serial reports touting the impact of recreational cannabis for pain [16]. Based on the evidence available, we continue to recommend only medical cannabis use for chronic pain unresponsive to other modalities [8].

Other Health Considerations and Cannabis

Cannabis currently remains on the list of banned performance enhancers in the Olympics [2], with calls on both sides of the divide regarding this issue. Whilst argument can be made for the potential deleterious effects of the drug on the health of athletes, there is no concrete evidence that performance of any kind, per se, is impacted positively by its use [2]. Some reports indicate that performance is actually reduced and subjects were noted to show more weakness with relation to endurance sports [2]. Blood pressures were reported to fall after chronic use, (115.8-107.9 mm) and diastolic (62.8-53.3 mm) pressures, in both cases p < 0.01, but increased fatigue, persistent tachycardia and impaired blood pressure response in users [2]. There was a uniform finding of increased perception of difficulty with exercising after use of the drug and measured performance was notably reduced [2]. The adverse effects appear to pale for medical cannabis as compared to the recreational counterpart, with incidence rates of cannabis-related emergencies as low as 8.06 per 10,000 person-years in medical cannabis users (95% CI: 4.8-13.6) [9]. Despite the above assertions, there is an abundance of top-flight athletes who confess using the drug recreationally and sometimes competitively remain [17,18]. We therefore recommend that individuals make decisions about whether or not this is worth the risk only after thorough evaluation of existing evidence.

We also took a look at cannabis use as a nutrient supplement. None of our studies hinted at a direct or indirect nutrient role of the plant, beyond the existence of an abundance of flavonoids [1,19,20]. In animal studies, particularly amongst ruminants, high supplemental protein and high levels of detergent fiber were noted [20]. We will not, in this study, extrapolate our findings in humans.

Recommendations and Further Studies

Cannabis-related cardiovascular disorders have seen increasing prevalence over the last decade [1]. This may require further investigation, as use prevalence is comparable to other substances of abuse. Guidelines for specific antidotes for cannabis and further legislature may need to be developed to manage these events [5]. For patients with pre-existing heart disease, it is recommended that risk calculators or better policy be devised to measure or mitigate the additional risk marijuana poses with use [5]. The same should be developed for young and healthy individuals who seek to engage in use [1]. It should be noted that cannabis has immune suppressive properties and hence may predispose users to higher risk of septicemia, with the respiratory system at particular risk, perhaps owing to the preferred route of recreational use [10].

Limitations

Our study faced a number of limitations. It has been reported that the plant may, in some context, be used to replace other psychoactive medications and may have a role in substance use treatment [21]. Our first limitation was that our study fell short of investigating this finding. Despite the growing concern for the side effects pointed out in the study, this may be another reason to consider the drug therapeutically. Secondly, our study did not place premium on distinguishing THC from CBD, which are distinct chemical components of cannabis. We did not make the distinction between other flavonoids, with chemicals numbering more than 500 [1]. Whilst cannabis itself is not approved for medical purposes, the FDA has approved some components for medical use [22]. The distinction may have helped provide more conclusive findings in our review. Thirdly, we based our interest on cardiovascular and mental health events and effects of marijuana. This limited our search options and narrowed our review base. Cannabis is known to affect several systems, which may interplay in ways yet to be delineated. Fourthly, we were interested in finding actual cardiovascular benefits of cannabis applicable in both clinical and non-clinical settings. The studies showed zero benefit in this regard, limiting much of the scope of our findings to negative effects. Finally, whilst we found documented evidence for mental health benefits and improvement in chronic pain [8], we found no research touting cannabis as the first-line agent for treating or managing any ailment. This, sadly, reduces the medical relevance of the drug compared with its popularity as a recreational agent.

## Conclusions

We were interested in investigating the physical benefits, cardiac and mental health benefits of cannabis, for medical and recreational purposes. We were also interested in finding out evidence of the side effects in these domains of health. Our study placed emphasis on non-mental health-related research, as evidenced by our search strategy. We found that there was no clear benefit of marijuana to heart health. Mental health benefits appeared to be route of administration, formulation and dose dependent. And other physical benefits appear to be limited to chronic pain patients. This implies that much of the famed benefits of the drug may only be anecdotal, at best. We recommend more studies be directed towards management of cannabis-induced cardiomyopathies and heart disease and ratification of guidelines for specific management. More attention should be paid to the mass education of these effects in the general population, including the seemingly young and healthy, as they have also been implicated in these adverse effects.
